# Food Safety Practices among Postnatal Mothers in Western Ghana

**DOI:** 10.1155/2020/8891605

**Published:** 2020-11-22

**Authors:** Stephen T. Odonkor, Napoleon Kurantin, Anthony M. Sallar

**Affiliations:** ^1^School of Public Service and Governance, Ghana Institute of Management and Public Administration, Accra, Ghana; ^2^School of Liberal & Social Sciences, Ghana Institute of Management and Public Administration, Accra, Ghana

## Abstract

Food safety has become a global issue due to the morbidity and mortality associated with it, particularly in developing countries. The objective of this community-based study is to examine food safety practices and its associated factors among postnatal mothers in the Western Region of Ghana. A cross-sectional survey study was conducted from August 1 2019 to January 31, 2020 from which data was obtained from the postnatal mothers (*N* = 300). The data was analysed using SPSS v.23. The findings suggest that majority (86%) of the respondents exhibited good food handling practices. Also, 66.3% and 91.7% of the respondents exhibited good food preparation and proper storage practices, respectively. Results also revealed that the odds of performing good handling practice among respondents within the age group of 36-45 years were five times higher compared to those within the age group of 18-25 years [OR = 4.92 (95% CI: 1.44–16.86), *p* = 0.011]. Moreover, respondents who had tertiary education qualifications were more likely to have proper food handling practices compared to those with primary education [OR = 0.27 (95% CI: 0.09–0.71), *p* = 0.009]. These findings provide useful insights for policy directions. The government of Ghana and other stakeholders should develop a communication strategy to increase and sustain publicity and education on food safety particularly to postnatal mothers and the citizenry in general.

## 1. Introduction

In recent times, food safety has become an issue of global attention particularly due to its significant link to public health and the need to minimise foodborne diseases [1]. Moreover, food safety is critical in sustaining food security in every nation [2]. One major global concern about unsafe food is its high probability of causing diseases which are usually termed as foodborne diseases. Foodborne diseases are significant causes of deaths and a major hindrance to physical productivity and socioeconomic development worldwide (WHO, 2015). Though the full scale of the global estimates of foodborne diseases is uncertain, it is estimated that generally, the discovered 31 worldwide foodborne hazards caused about 600 million foodborne illnesses and 600,000 deaths in 2010 alone [3]. According to the World Health Organization, Africa and Asia are perceived to have the highest incidence of foodborne disease. There are over 250 foodborne diseases [4, 5]. A study in Ghana using data from 2009 to 2013 revealed cholera as the predominant foodborne disease [5].

Unsafe food handling practices tend to increase the transmission of foodborne diseases [1]. A food handler is anyone who manipulates food or anything that has a high probability of coming into contact with food or foodstuffs [6]. Food handlers play a vital role in ensuring food safety and as a result, the global concern towards this issue significantly involves them as well [7]. About 10% to 20% of foodborne disease occurrences have been attributed to contamination by food handlers [8]. It is well recognized that food handlers in homes, who are usually mothers, are important last line of defence against foodborne disease outbreak. Thus, their relevance in ensuring food safety in homes cannot be underestimated ([1]; Rao et al., 2007; [9]). Other studies have further highlighted that over 50% of foodborne diseases come from domestic homes [10, 11], which is aggravated by lack of requisite food safety practices and knowledge [11, 12]. Hence, investigation of food safety practices among mothers is highly needed. For postnatal mothers, food safety practices are extremely relevant not only for their personal health but also for good child care. Children have an undeveloped immune system and, as a result, are at higher risks of acquiring foodborne diseases than adults. Statistics show that out of the 2.2 million people who die annually due to foodborne diseases in developing countries, an estimated 1.9 million are children [12]. Additionally, 40% of foodborne disease burden in 2010 were among children under 5 years of age, globally (WHO, 2015).

There have been studies conducted to investigate food safety practices among street vendors and other institutional centres in Ghana [13–15], but in relation to mothers, such studies are almost elusive despite the reported significant contribution of mothers to the increasing rate of foodborne diseases. Thus, food safety practices and its associated factors among mother, including postnatal mothers, are poorly understood in Ghana. Investigating food safety practices among food handlers is very necessary for guiding policymakers to put up structures that will minimize the foodborne disease outbreak. In a recent study in Ethiopia, half of the participants had a good self-reported food safety practice, and the other half's level was poor [1]. In another study, mothers had good knowledge on personal hygiene and food poisoning, but their food handling practices were poor. These findings showed that good knowledge of food safety does not necessarily translate into good attitudes and practices. Knowledge, attitudes, and practices of food safety are important in preventing food poisoning [16]. Hence, the objective of this community-based study is to examine food safety practices and its associated factors among postnatal mothers in the Western Region of Ghana.

## 2. Materials and Methods

### 2.1. Study Site Description

**Figure 1:**
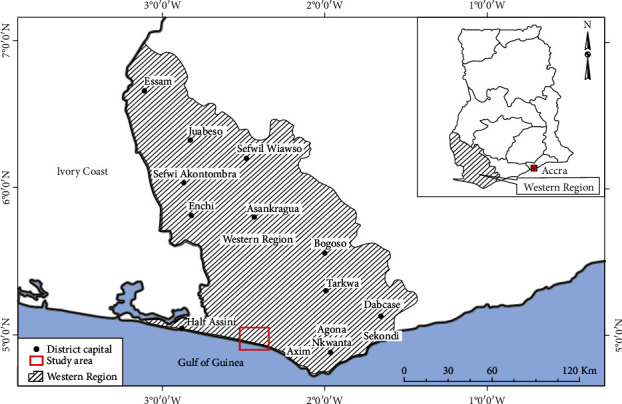
Map of the Western Region.

The Western Region of Ghana is the home to Ghana's only twin city: Sekondi-Takoradi. The region can be found in the southern part of Ghana (Figure 1). It is sandwiched between Ivory Coast to the west and the central region to the east. The Western region of Ghana has a coast line as its south boundary. The region occupies a land size of 23,921 sq. km. This accounts for about 10% of Ghana's total land area. The population of the region as of 2019 is estimated to be 2,165,241 out of which females constitute 1,100,443 (GSS, 2020). There are 13 districts in the region, and the population density is 80.5 sq. km. Females have lower literacy rate (47.9%) in the region as compared to males (68.0%).

### 2.2. Research Design

The study employed a cross-sectional design. Self-administered questionnaires were used to obtain data on food safety practices and its associated factors among postnatal mothers in the Western Region of Ghana. The study was conducted from August 1, 2019 to January 31, 2020. Questionnaires were self-administered and took an average of 28 minutes to complete. The average margin of error is 95% confidence interval.

### 2.3. Sampling Technique

Data were obtained from a regionally representative survey of postnatal mothers (*N* = 300). The study utilized a stratified sampling technique. The region was demarcated into 3 zones: southern-western belt, middle belt, and northwestern belt. The study utilized a stratified sampling technique to obtain the required number of respondents from hospitals within the three (3) demarcated zones. Thus, in selecting the respondents, sampling proportionate to size was used to determine the number of postnatal mothers to be interviewed from each category of the zones. All postnatal mothers present in the demarcated zone were considered for the study.

### 2.4. Sample Size

The sample size for the study was determined using the Miller and Brower formula (*n* = *N*/1 + *N*[*α*)^2^]) for the sample size estimation [17]. The formula was thus employed to determine the sample taken from each hospital. In the formulae: *n* = samplesize, *N* = totalpopulation, and*α* = marginoferror, nonresponses and inappropriately filled questionnaires were accounted for by increasing the minimum sample size by 10%. Thus, a total of 316 questionnaires were distributed for the study. However, 300 were completely filed and returned. This represents a 95% response rate.

### 2.5. Data Collection Instrument

A standardized questionnaire was used to obtain the data. Field inspection of the questionnaire data was carried out days after the interview were conducted, and any errors were immediately verified and corrected. The survey instrument comprised of 21 questions in four (4) thematic areas, namely, demographic characteristics, food handling practices among postnatal mothers, food storage practices among postnatal mothers, and food preparation practices among postnatal mothers. The questionnaire was designed and administered in the English language. It took approximately 25–30 minutes to complete the instrument. Six experts in social sciences measurement and evaluation determined face validity of the instrument. The average overall face validity was equal to 95%. The study used Cronbach's alpha test formula to test the reliability of the standard questionnaire. The test yielded a reliability coefficient of 0.8. Cronbach's alpha test assesses the internal consistency of a set of scale of or items to ensure that they are all consistent in measuring the same attributes under study.

### 2.6. Ethical Considerations and Data Handling

Ethical clearance was obtained from the School of Public Service and Governance (SPSG) Ethics Review Committee of the Ghana Institute of Management and Public Administration. Prior to the data collection, respondents' written and verbal consent was sought. The data were entered into an excel spreadsheet and later exported to SPSS version 23 and coded for analysis. Descriptive statistics and inferential statistical methods were used to describe the variables of interest.

## 3. Results and Discussion

### 3.1. Results

Table 1 shows the demographic characteristics of the respondents. It can be seen from the table that more than half (59%) of the respondents were within the 26-35 years age group. Majority of the respondents (65.3%) had an average monthly income of GHC 600. Almost all of the respondents (96%) were married whilst less than 1% of them were divorced. Majority of the respondents (88%) attained the primary level of education, and also 6.7% had completed tertiary education. Majority (69.7%) of the respondents had given birth to three children.

Table 2 presents food handling practices among respondents. Almost all (99.3%) of the respondents agreed that washing hands before handling food reduces the risk of food contamination. Majority (82.75) of the respondents also disagreed that it is necessary not to handle food during infectious disease of the skin. Moreover, 97.3% of the respondents agreed that there is a need to check for expiry dates especially on canned foods before consumption. Most of the respondents (84.3%) agreed that using gloves while handling food reduces the risk of food contamination.

Table 3 shows the food preparation practices among the respondents. Almost all of the respondents, 99.3%, agree that washing hands before handling food reduces the risk of food contamination. More than half of the respondents also agree that it is not necessary to handle food during infectious disease of the skin. Moreover, majority of the respondents 97.3% agree that there is a need to check for expiry dates especially on canned foods before consumption to help avoid foodborne disease. Most of the respondents, 84.3%, agree to the fact that using gloves while handling food reduces the risk of food contamination.

Table 4 shows the food storage practices among the respondents. Almost everyone (96.3%) purchases frozen meat at the end of their shopping trip. Also, a majority (95%) of the respondents put food that will be consumed in 3-4 hours, on the table or the counter and reheat it before consumption. Almost all of the respondents (87.7%) indicated that leftovers should not be kept in a fridge for more than 2 days.

**Figure 2:**
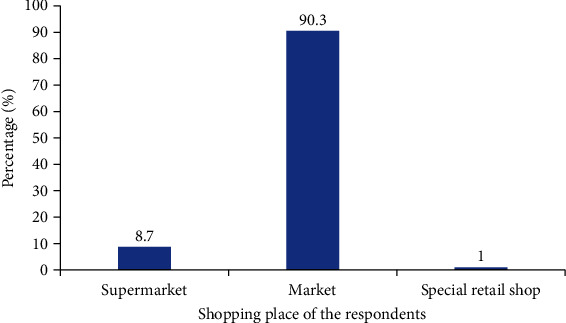
Shopping place of the respondents.

Figure 2 shows the places the respondents shop for their food preparation. Almost all (90.3%) of the respondents did their shopping at the market, while 8.7% shopped at the super market and only 1% at the special retail shop.

**Figure 3:**
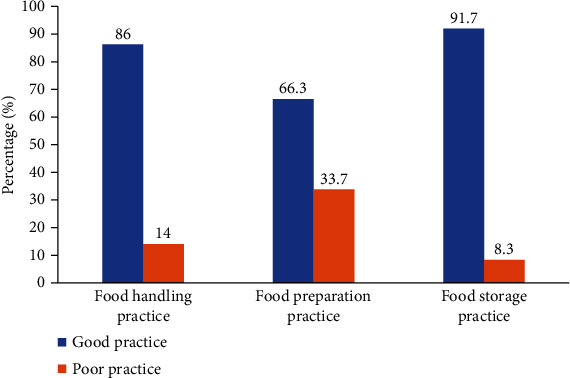
Food handling, preparation, and storage practices among respondents.

Figure 3 presents food handling, preparation, and storage practices among respondents. The findings suggest that majority (86%) of the respondents exhibited good food handling practices. It was revealed that 66.3% of the respondents had good food preparation practices. The findings also suggest that majority (91.7%) of the respondents were storing food properly.

Table 5 shows the association between respondent's food handling practices and demographic characteristics. The logistic regression analysis revealed that the odds of performing good handling practice among respondents within the age group of 36-45 years were five times higher compared to those within the age group of 18-25 years [OR = 4.92 (95% CI: 1.44–16.86), *p* = 0.011]. Moreover, respondents who had tertiary education qualification were more likely to have proper food handling practices compared to those with primary education [OR = 0.27 (95% CI: 0.09–0.71), *p* = 0.009]. Respondents who had three children were thrice more likely to have proper food handling practices [OR = 2.86 (95% CI: 1.09–7.49), *p* = 0.033] compared with those who had one child.

Table 6 shows the association between respondent's food preparation practices and demographic characteristics. The logistic regression analysis revealed that mothers whose monthly income were GHC 600-1000 were two times more likely to have good hygienic food preparation practices compared to those with less than GHC 500 as monthly income [OR = 2.45 (95% CI: 1.44–4.17), *p* = 0.001].

Table 7 shows the association between respondent's food storage practices and demographic characteristics. The logistic regression analysis indicates that respondents who were not married were less likely to have good food storage practices compared to those who were married [OR = 0.06 (95% CI: 0.02–0.21), *p* < 0.001]. Also, mothers who had secondary and tertiary education were less likely to have good food storage practices compared to those with primary education [OR = 0.01 (95% CI:0.003–0.05), *p* < 0.001] and [OR = 0.05 (95% CI:0.02–0.17), *p* < 0.001], respectively; however, the odds of having good food preparation practice among mothers who had three children were elevenfold higher as compared to those with one child [OR = 10.98 (95% CI: 3.25–37.13), *p* < 0.001].

### 3.2. Discussion

The present study investigated food safety practices and its associated factors among postnatal mothers in the Western Region of Ghana. Findings of this study will help to inform policy decisions that will aid improving food safety practices of postnatal mothers in this region.

Food safety practices of the participants were investigated in three categories including food handling practices, food preparation/personal hygiene practices, and food storage practices. The postnatal mothers demonstrated good self-reported knowledge on food handling and food storage practices with a passing rate of 86% and 91.7%, respectively. However, the postnatal mothers had relatively, moderate self-reported knowledge on safe food preparation/personal hygiene practices with a passing rate of 66.3%. These results are higher compared to studies among mothers in the Northeast Ethiopia [1], Saudi Arabia [12], and Miami, Florida (Trepka, 2007), as well as studies among food vendors in Nigeria [18, 19] and Ghana [20].

Though generally, respondents had good self-reported knowledge on food safety practice scores, and there were certain specific noteworthy findings. First of all, regarding food handling practices, 82.7% of the postnatal mothers disagreed “it is not necessary to handle food during infectious disease of the skin.” It signified that this these mothers either do not see anything wrong with handling food when the handler has a skin infection or have not been exposed to education on the possible crosscontamination that could occur in such a scenario. One of the ways to avoid foodborne bacteria illnesses or diseases is to prevent individuals with skin disease or lesions from handling food [21, 22]. Thus, there is a gap in the knowledge of the postnatal mothers in the Sekondi-Takoradi Metropolis in this regard.

Furthermore, most of the respondents were of the notion that it was not always necessary to keep finger nails short and clean during food preparation as 80.4% of the postnatal mothers indicated that this can be done sometimes. However, the World Health Organization [23] emphasizes the need for food handlers to always keep their nails short and clean. It is still dangerous to manipulate foodstuffs during food preparation even with long but clean nails because Rane [24] revealed that pathogens such as *Salmonella,* nontyphi salmonellae, *Campylobacter*, and *E*. coli can inhabit fingertips for varied periods of time. Thus, it is necessary for food handlers and food vendors to keep their finger nails short and clean to prevent them from being transmitting agents of pathogens [25].

In Ghana, there are three places to shop for food items, food ingredients, or ready to eat meals, and these are supermarkets, traditional markets, and special retail shops. A large majority of the postnatal mothers revealed the traditional markets as their shopping destinations as opposed to the supermarket and special retail shops. There are several likely explanations to this observation. Firstly, the traditional food retail outlet remains significant in Ghana's agri-food system [26, 27]. Meng et al., (2014), revealed through their findings that these traditional open-air markets still dominate the Ghana food retailing system. Moreover, such markets provide large households with locally produced foods at a cost that favours individuals with low incomes and low educational background [27]. These corroborate with the income and educational profile of most respondents in the present study. It is worth noting that other factors such as easy availability and accessibility to the traditional markets as compared to the other markets could be possible reasons as well.

Bivariate multilogistic analysis revealed associated factors linked to food handling practices, food preparation practices, and food storage practices among the respondents. There was no significant association between level of food handling practices and respondents' age group, monthly income, marital status, occupational status, type of delivery, and educational level. This was consistent with a similar study by Aung et al. [28], where there was no significant association between the age group, income level, educational level, and food handling practice of the respondents. However, in the present study, there was an association between food handling practice and respondents' religion, ethnicity, place of residence, occupational status, and number of children. Regarding of food preparation practices, there were only significant associations between ethnicity and number of children. For food storage practices, findings revealed marital status, religion, place of residence, occupational status, type of delivery, educational level, and number of children as significant associating factors.

The above findings revealed few inconsistencies in associated factors of food handling, preparation, and storage practices among the postnatal mothers in the Western Region of Ghana. For instance, the educational level was a significant associated factor of food storage practices among the respondents but was insignificant with regards to their food handling and food preparation practices. Regardless, it would still be less productive for appropriate stakeholders and policymakers to increase the literacy rate of the postnatal mothers in an attempt to improve their food storage practices. This is because results showed that respondents with primary education were more likely to have hygienic food storage practices as compared to those with secondary and tertiary educational background. In contrast, other studies reported that higher educational background of the respondents was associated with good food safety practices [1, 12, 29, 30]. The number of children among the respondents was the only consistent associated factor across the three categories of food safety practices. In summary, associated factors of food safety practices among the postnatal mothers in the Western Region of Ghana require further investigations to fully understand or explain.

## 4. Conclusion

The self-reported food safety practices among the respondents were generally good. However, the respondents indicated higher knowledge in hygienic food handling and food storage practices as compared to food preparation practices. Specifically, most respondents did not agree on keeping short and clean nails always when preparing food and also not handling food when the handler has a skin infection or lesion. Thus, there should policies that should be made towards enlightening postnatal mothers in the Western Region of Ghana in this regard. The present study found out significant associated factors of food safety practices among the respondents. However, the inconsistencies in the findings require further investigations to clearly understand and explain.

## Figures and Tables

**Table 1:** Demographic characteristics of respondents.

Variable	Frequency	Percentage
Age group		
18-25 years	44	14.7
26-35 years	177	59.0
36-45 years	71	23.7
Above 45 years	8	2.6
Monthly income		
< Ghc500	83	27.7
Ghc600-1000	196	65.3
Ghc1001-1500	12	4.0
> Ghc1500	9	3.0
Marital status		
Married	288	96.0
Single	11	3.7
Divorced	1	0.3
Religion		
Christianity	293	97.7
Islamic	7	2.3
Type of delivery		
Normal	290	96.67
Caesarean	10	3.33
Educational level		
Primary	264	88.0
Secondary	16	5.3
Tertiary	20	6.7
Number of children		
One	33	11.0
Two	52	17.3
Three	209	69.7
Four	4	1.3
Five	2	0.7

**Table 2:** Food handling practices among the respondents.

Variable	Agree, no. (%)	Disagree, no. (%)	Do not know, no. (%)
Washing hands before handling food reduces the risk of food contamination	298 (99.3)	2 (0.7)	0 (0)
Using gloves while handling food reduces the risk of food contamination	253 (84.3)	39 (13.0)	8 (2.7)
Improper cleaning and sanitization of utensils increases the risk of food contamination	256 (85.3)	43 (14.3)	1 (0.4)
Reheating cooked foods contributes to food contamination	37 (12.3)	258 (86.0)	5 (1.7)
Crosscontamination occurs when microorganisms are transferred by the food handler's hand	290 (96.7)	8 (2.6)	2 (0.7)
It is necessary to handle food during infectious disease of the skin	51 (17.0)	248 (82.7)	1 (0.3)
Need to check the expiry date of canned food	292 (97.3)	6 (2.0)	2 (0.7)
Read the instruction for use and for preservation written on the pack	293 (97.7)	6 (2.0)	1 (0.3)
Check if a canned food is dented before you buy	286 (95.3)	11 (3.7)	3 (1.0)

**Table 3:** Food preparation practices among the respondents.

Variable	Always, no. (%)	Often, no. (%)	Sometimes, no. (%)	Rarely, no. (%)	Never, no. (%)
Cover dustbins where food is prepared	292 (97.3)	3 (1.0)	4 (1.3)	1 (0.4)	0 (0)
Availability of potable water for cooking	292 (97.2)	1 (0.3)	7 (2.4)	0 (0)	0 (0)
Use clean water to wash hands and plates	296 (98.7)	2 (0.7)	1 (0.3)	1 (0.3)	0 (0)
Cover hair when preparing food	277 (92.3)	6 (2.0)	11 (3.7)	6 (2.0)	0 (0)
Ensure that fingernails are kept short and clean when cooking	49 (16.3)	7 (2.3)	241 (80.4)	3 (1.0)	0 (0)
Cover mouth with handkerchief when sneezing or yawning	279 (93.0)	4 (1.33)	16 (5.33)	1 (0.33)	0 (0)
Cook when have foodborne or airborne disease	77 (25.7)	9 (3.0)	21 (7.0)	13 (4.3)	180 (60.0)
Wash hands with soap and water after using the washroom	274 (91.3)	8 (2.70	3 (1.0)	9 (3.0)	6 (2.0)
Wash hands with soap before cooking	288 (96.0)	9 (3.0)	3 (1.0)	0 (0)	0 (0)
Clean foodstuffs before cooking them	286 (95.3)	2 (0.7)	3 (1.0)	2 (0.7)	7 (2.3)

**Table 4:** Food storage practices among the respondents.

Variable	Frequency	Percentage
Purchase frozen/refrigerated food when shopping		
At the end of the shopping trip	289	96.3
At the beginning of the shopping trip	4	1.3
Whenever, does not matter	7	2.4
Prepared food that will be consumed 3-4 hours later		
Put it on the table/counter, then reheat before consumption	285	95.00
Put it in the refrigerator, then reheat before consumption	7	2.3
Store it in the microwave oven or regular oven, then reheat before consumption	8	2.7
Eggs can contain germs. How to store raw eggs at home		
Wash them with soap and water and store in the refrigerator	273	91.0
Wipe with a dry cloth and store in the refrigerator	24	8.0
Store directly in the refrigerator then wash your hands after touching them	3	1.0
Leftovers should be kept in the fridge		
No more than 2 days	263	87.7
No more than 5 days	29	9.6
As long as they smell good, we can eat them	8	2.7
Most dangerous way to thaw frozen meat		
Thaw in the refrigerator	265	88.3
Thaw on the kitchen counter	16	5.3
Use the microwave oven and then cook immediately	15	5.0
Thaw under cold water	4	1.4
Reheated leftovers (in microwave) still not eaten completely		
Discard them immediately	274	91.3
Put in the refrigerator immediately and reheat again before consumption	5	1.7
Leave on the kitchen counter and reheat again before consumption	21	7.0

**Table 5:** Association between food handling practices and respondent's demographic characteristics.

Variable	Food handling practices		*χ* ^2^	*p* value	OR (95% CI), *p* value
	Poorpractice = 42 *n* (%)	Properpractice = 258 *n* (%)			
Age group			8.69	0.034	
18-25 years	10 (23.8)	34 (13.2)			
26-35 years	28 (66.7)	149 (57.8)			1.57 (0.69–3.53), 0.280
36-45 years	4 (9.5)	67 (25.9)			4.93 (1.44–16.87), 0.011
Above 45 years	0 (0.0)	8 (3.1)			1 (empty)
Monthly income			6.90	0.075	
< Ghc500	15 (35.8)	68 (26.4)			
Ghc600-1000	21 (50.0)	175 (67.8)			1.84 (0.89–3.77), 0.097
Ghc1001-1500	3 (7.1)	9 (3.5)			0.66 (0.16–2.74), 0.569
> Ghc1500	3 (7.1)	6 (2.3)			0.44 (0.09–1.93), 0.283
Marital status			1.82	0.402	
Married	39 (92.9)	249 (96.5)			
Single	3 (7.1)	8 (3.1)			0.42 (0.10–1.64), 0.211
Divorced	0 (0.0)	1 (0.4)			1 (empty)
Type of delivery			2.19	0.138	
Normal	39 (92.9)	251 (97.3)			
Caesarean	3 (7.1)	7 (2.7)			0.36 (0.09–1.46), 0.154
Educational level			7.85	0.020	
Primary	33 (78.6)	231 (89.5)			
Secondary	2 (4.8)	14 (5.4)			1.00 (0.22–4.59), 1.000
Tertiary	7 (16.7)	13 (5.1)			0.27 (0.09–0.71), 0.009
Number of children			28.92	<0.001	
One	7 (16.7)	26 (10.1)			
Two	15 (35.7)	37 (14.3)			0.66 (0.24–1.86), 0.435
Three	18 (42.8)	191 (74.0)			2.86 (1.09–7.49), 0.033
Four	0 (0.0)	4 (1.6)			1 (empty)
Five	2 (4.8)	0 (0.0)			1 (empty)

**Table 6:** Association between food preparation practices and respondent's demographic characteristics.

Variable	Food preparation practices		*χ* ^2^	*p* value	OR (95% CI), *p* value
	Unhygienic = 101, *n* (%)	Hygienic = 199, *n* (%)			
Age group			5.38	0.146	
18-25 years	14 (13.9)	30 (15.1)			
26-35 years	55 (54.5)	122 (61.3)			1.04 (0.51–2.11), 0.924
36-45 years	31 (30.7)	40 (20.1)			0.60 (0.27–1.33), 0.208
Above 45 years	1 (0.9)	7 (3.5)			3.27 (0.37–29.17), 0.289
Monthly income			11.13	0.011	
< Ghc500	40 (39.6)	43 (21.6)			
Ghc600-1000	54 (53.4)	142 (71.4)			2.45 (1.44–4.17), 0.001
Ghc1001-1500	4 (4.0)	8 (4.0)			1.86 (0.52–6.66), 0.340
> Ghc1500	3 (3.0)	6 (3.0)			1.86 (0.44–7.94), 0.402
Marital status			0.54	0.762	
Married	97 (96.0)	191 (96.0)			
Single	4 (4.0)	7 (3.5)			0.89 (0.25–3.11), 0.854
Divorced	0 (0.0)	1 (0.5)			1 (empty)
Type of delivery			0.19	0.666	
Normal	97 (96.0)	193 (97.0)			
Caesarean	4 (4.0)	6 (3.0)			0.75 (0.21–2.73), 0.667
Educational level			2.74	0.254	
Primary	89 (88.1)	175 (88.0)			
Secondary	3 (3.0)	13 (6.5)			2.20 (0.61–7.93), 0.227
Tertiary	9 (8.9)	11 (5.5)			0.62 (0.25–1.56), 0.310
Number of children			14.69	0.005	
One	14 (13.9)	19 (9.5)			
Two	26 (25.7)	26 (13.1)			0.74 (0.31–1.77), 0.496
Three	58 (57.4)	151 (75.9)			1.92 (0.90–4.08), 0.90
Four	1 (1.0)	3 (1.5)			2.21 (0.21–23.56), 0.511
Five	2 (2.0)	0 (0.0)			1 (empty)

**Table 7:** Association between food storage practices and respondent's demographic characteristics.

Variable	Food storage practices		*χ* ^2^	*p* value	OR (95% CI), *p* value
	Poorstorage = 25, *n* (%)	Goodstorage = 275, *n* (%)			
Age group			4.87	0.182	
18-25 years	5 (20.0)	39 (14.2)			
26-35 years	18 (72.0)	159 (57.8)			1.13 (0.39–3.24), 0.817
36-45 years	2 (8.0)	69 (25.1)			4.42 (0.82–23.88), 0.084
Above 45 years	0 (0.0)	8 (2.9)			1 (empty)
Monthly income			1.62	0.655	
< Ghc500	9 (36.0)	74 (26.9)			
Ghc600-1000	15 (60.0)	181 (65.8)			1.47 (0.62–3.50), 0.387
Ghc1001-1500	1 (4.0)	11 (4.0)			1.34 (0.15–11.61), 0.792
> Ghc1500	0 (0.0)	9 (3.3)			1 (empty)
Marital status			31.98	<0.001	
Married	19 (76.0)	269 (97.8)			
Single	6 (24.0)	5 (1.8)			0.06 (0.02–0.21), <0.001
Divorced	0 (0.0)	1 (0.4)			1 (empty)
Type of delivery			36.15	<0.001	
Normal	19 (76.0)	271 (98.6)			
Caesarean	6 (24.0)	4 (1.4)			0.05 (0.01–0.18), <0.001
Educational level			106.23	<0.001	
Primary	7 (28.0)	257 (93.5)			
Secondary	11 (44.0)	5 (1.8)			0.01 (0.003–0.05), <0.001
Tertiary	7 (28.0)	13 (4.7)			0.05 (0.02–0.17), <0.001
Number of children			50.12	<0.001	
One	7 (28.0)	26 (9.4)			
Two	9 (36.0)	43 (15.6)			1.29 (0.43–3.87), 0.654
Three	5 (20.0)	204 (74.2)			10.98 (3.25–37.13), <0.001
Four	3 (12.0)	1 (0.4)			0.09 (0.01–1.00), 0.050
Five	1 (4.0)	1 (0.4)			0.27 (0.01–4.87), 0.374

## Data Availability

The data used to support the findings of this study are included within the article.
